# Leukemoid Reaction in a Patient With Severe COVID-19 Infection

**DOI:** 10.7759/cureus.13598

**Published:** 2021-02-27

**Authors:** Kidist Tarekegn, Ana Colon Ramos, Harry G Sequeira Gross, Ming Yu, Ilmana Fulger

**Affiliations:** 1 Internal Medicine, St. Barnabas Hospital Health System, The Bronx, USA; 2 Hematology-Oncology, St. Barnabas Hospital Health System, The Bronx, USA

**Keywords:** leukemoid reaction, covid-19, leukocytosis

## Abstract

Leukemoid reaction is a rare clinical condition defined by marked leukocytosis with predominantly mature neutrophils. It is an uncommon condition with reported incidence of less than 1% in hospitalized patients. The major causes of leukemoid reaction are severe infection (clostridium difficile colitis, tuberculosis, or severe shigellosis), malignancies, intoxication, and severe hemorrhage. This article presents a case report of a 64-year-old female with severe COVID-19 infection who presented with marked leukocytosis. After extensive workup to exclude hematologic malignancy, the patient was diagnosed to have a leukemoid reaction.

## Introduction

Leukemoid reaction is an uncommon clinical condition defined as leukocytosis >50,000 cells/mL with predominantly mature neutrophils and accompanying left shift [1,2]. It is known as a rare manifestation of systemic inflammation or stress and has been reported in malignant and non-malignant disease processes [1,3]. Coronavirus disease (COVID-19) has wide spectrum of clinical manifestations involving multiple organ systems. COVID-19 infection clinical presentation can range from asymptomatic disease to severe sepsis with multiple organ failure [4]. Its most common clinical features include fever, fatigue, cough, and diarrhea. The specific laboratory abnormalities of COVID-19 infection include lymphopenia, elevated lactate dehydrogenase (LDH), ferritin, D-dimer and C-reactive protein (CRP), and higher levels of interleukins (IL-2, IL-7 and IL-10) [4]. A meta-analysis concluded that leukocytosis and elevated C-reactive protein on arrival may predict poor outcomes in hospitalized patients in contrast with leukopenia which was associated with better outcomes [5,6]. As per our extensive literature review, this is the second case report of hyperleukocytosis with neutrophilia (leukemoid reaction) in COVID-19 [7]. Here, we present a case of marked leukocytosis and severe organ dysfunction in the setting of COVID-19 infection, which after an extensive workup to exclude hematologic malignancy was attributed to leukemoid reaction.

## Case presentation

A 64-year-old female patient presented to the emergency department with worsening of shortness of breath and new onset diarrhea. A week prior, the patient tested positive for COVID-19 infection at an urgent care center and was prescribed Cefdinir and Azithromycin. At presentation, the patient complained of fever, shortness of breath, dry cough, and diarrhea. On examination at the emergency department, she was febrile (temperature: 100.9° F), hypoxic (oxygen saturation: 88% on room air), tachypneic (respiratory rate: 24/minute), and tachycardic (heart rate: 97/minute). She was in respiratory distress. She was noted to be obese with BMI of 30.1 kg/m^2^. The rest of the physical examination was unremarkable.

**Figure 1:**
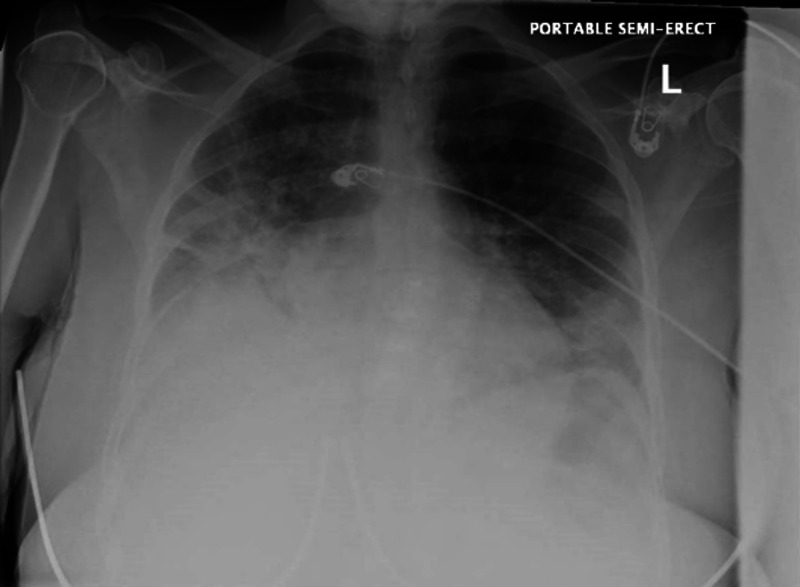
Chest X-ray at presentation to the emergency department showing diffuse predominantly basal and peripheral opacities

Initial investigation at the emergency department showed marked leukocytosis with left shift (WBC 46.8 x 10^3^/mL with absolute neutrophil count [ANC] 32.09 x 10^3^/mL), normocytic anemia (hemoglobin 6.5 gm/dl, mean corpuscular volume [MCV] 88.6, mean corpuscular hemoglobin [MCH] 29.7), thrombocytosis (platelet count 412 x 10^3^/mL), elevated inflammatory markers (lactate dehydrogenase [LDH]: 519 IU/L, C-reactive protein: 33.71 mg/dl, erythrocyte sedimentation rate [ESR]: >100 mm/hr and ferritin: 2257.0 ng/ml), elevated D-Dimer (1.46 mg [FEU]/L), and bilateral predominantly basal and peripheral infiltrates with right pleural effusion on chest X-ray (Figure 1). Oxygen saturation improved to 96% on oxygen 6L/minute via nasal canula. She was admitted to the general medical floor under the impression of acute hypoxemic respiratory failure secondary to COVID-19 infection. At this stage, the patient was started on steroid (Methylprednisolone), antibiotics (Vancomycin, Azithromycin and Cefepime), Hydroxychloroquine, and Enoxaparin, then Apixaban (for DVT prophylaxis) as per the standard of care at the time. On the second day of admission, the patient’s hemoglobin dropped to 5.3 gm/dl. However, she refused transfusion because of religious reasons and she was later started on IV iron infusion and weekly erythropoietin.

**Figure 2:**
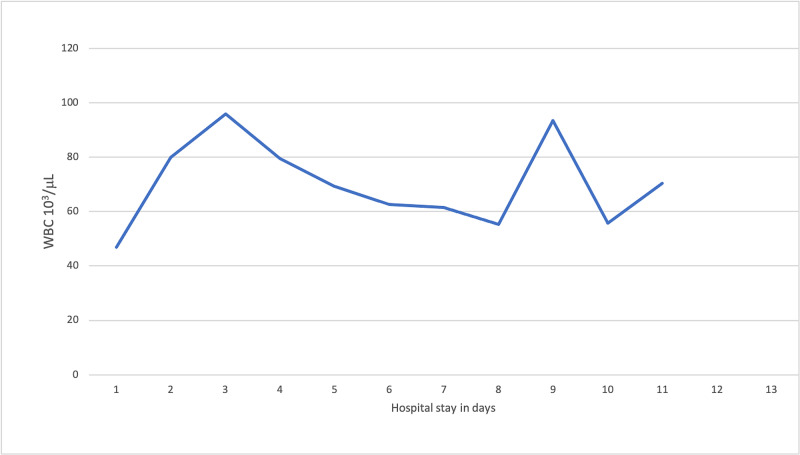
Evolution of white blood cell (WBC) count during hospital course

Follow-up laboratory workup showed persistent leukocytosis (Figure 2), elevated Interleukin-6 (19 pg/ml), and inflammatory markers (Table 1). Subsequently, the patient was given tocilizumab. However, her hypoxemia worsened despite the increment of supplemental oxygen and she was urgently intubated, placed on mechanical ventilator and transferred to medical ICU. On day 6 of hospitalization, the patient was started on remdesivir. However, the patient continued to be in critical condition. This patient was managed in our hospital at the beginning of the pandemic. The current treatment protocol for COVID-19 infection in our hospital includes early treatment with dexamethasone, remdesivir, convalescent plasma and in some cases monoclonal antibodies.

**Table 1:** Evolution of laboratory parameters during hospital course WBC: White blood cells; LDH: Lactate dehydrogenase; ESR: Erythrocyte sedimentation rate.

	Day 1	Day 2	Day 3	Day 4	Day 6	Day 7	Day 8	Day 9	Day 10	Day 11	Day 12	Day 13
WBC (10^3^/mL)	46.8	80	95.9		79.4	69.2	62.7	61.5	55.4	93.5	55.7	70.3
Neutrophil %	68.6	74.8	67.1		77.4	77	82.1	80.6	80.8	89	84.8	86.2
Hemoglobin (gm/dl)	6.5	6.3	6		6.3	6.6	7.2	7	7.3	6.2	7	6.5
Platelet (10^3^/mL)	412	380	347		102	40	15	51	11	22	9	14
LDH (IU/L)	579	453	522	512	713	650	721	961	964			1347
Ferritin (ng/ml)	2257	1994	2541	3319	3111	4006	2776	3036	2617			>7500
C-Reactive protein (mg/dl)	33.71			17.05	3.59	3.3	3.39	7.19	11.92			
ESR (mm/hr)			>100									
Interleukin-6 (pg/ml)		19	34		2743	1924						
D-Dimer [mg (FEU)/L]	1.46	2.69	5.02		22.39	17.94	20.84	>35.20	>35.20			>35.20

The next follow-up laboratory investigation showed persistent leukocytosis, thrombocytopenia (trending down starting from day 4 with nadir 9000/mL on day 12), abnormal coagulation profile (Prolonged prothrombin time [PT], partial thromboplastin time [PTT] and international normalized ratio [INR]), normal fibrinogen level, and negative heparin-induced platelet antibody. Moreover, the patient had negative respiratory, blood and urine cultures. Peripheral blood smear showed neutrophilia and left shift with many bands with toxic granulations, metamyelocytes and myelocytes. There were moderate smudge cells, occasional large platelets and atypical lymphocytes. There were no blasts and no increase in basophils and eosinophils was noted. Further workup of the persistent leukocytosis with peripheral blood flow cytometry was negative for abnormal myeloid maturation or any immunophenotypic evidence of a lymphoproliferative disorder. Moreover, fluorescence in situ hybridization (FISH) was negative for BCR/ABL, JAK2, CALR and MPL mutations. Subsequently, the patient was diagnosed to have leukemoid reaction secondary to COVID-19 infection. The patient remained in critical condition and passed away in the hospital on day 13.

## Discussion

This report presents a case of leukemoid reaction in a patient with severe coronavirus disease (COVID-19). Leukemoid reaction is defined as leukocytosis (predominantly mature neutrophils) greater than 50,000 cells/mL with accompanying left shift [1,2]. Leukemoid reaction is in general an uncommon condition with an incidence of less than 1% in hospitalized patients [3]. The major causes of leukemoid reaction include severe infection (clostridium difficile colitis, tuberculosis, severe shigellosis, etc.), malignancies, intoxication, severe hemorrhage, or acute infection [2].

Coronavirus disease (COVID-19) has a wide spectrum of clinical manifestations ranging from asymptomatic disease to severe infection involving multiple organ systems. Hematologic manifestations of COVID-19 include lymphopenia, leukocytosis, coagulopathy and elevation of inflammatory markers (elevated LDH, ferritin, and CRP) [4].

In this case, the patient had persistent leukocytosis that was greater than 50,000 cells/mL with left shift. A peripheral blood flow cytometry and FISH for BCR/ABL, JAK2, CALR and MPL mutations were done to rule out myeloproliferative disorders and there were no immunophenotypic evidence of a lymphoproliferative disorder on flow cytometry and BCR/ABL FISH. Workup for other potential infectious causes was unremarkable as initial routine blood and respiratory cultures were negative. Moreover, Clostridium difficile colitis was ruled out with a negative stool PCR test. According to the scientific literature, leukocytosis and elevated C-reactive protein were found to be predictors of poor outcome in patients with COVID-19 [6,8]. Similarly, this case is an example where the patient presented with elevated WBC and C-reactive protein and had poor outcome.

## Conclusions

Although leukemoid reaction is an uncommon diagnosis in hospitalized patients, the case presented here shows a leukemoid reaction in a patient with severe COVID-19 infection. Moreover, the persistently elevated leukocytosis and C-reactive protein were shown as predictors of poor prognosis in this case.
